# Hydrogen storage of Li_4_&B_36_ cluster

**DOI:** 10.1038/s41598-018-20452-8

**Published:** 2018-01-31

**Authors:** Jiguang Du, Xiyuan Sun, Li Zhang, Chuanyu Zhang, Gang Jiang

**Affiliations:** 1 College of Physical Science and Technology, Sichuan University, Chengdu, 610064 China; 2 College of Science, Sichuan Agricultural University, Ya’an, 625014 China; 3 Institute of Atomic and Molecular Physics, Sichuan University, Chengdu, 610065 China; 4 Department of Physics, Chengdu University of Technology, Chengdu, 610059 China

## Abstract

The Saturn-like charge-transfer complex Li_4_&B_36_, which was recently predicted with extensive first-principles theory calculations, were studied as a candidate for hydrogen storage material in the present work. The bonding characters of Li-B, B-B and Li-H_2_ bonds were revealed by the quantum theory of atoms in molecules (QTAIM). Each Li atom in Li_4_&B_36_ cluster can bind six H_2_ molecules at most, which results into the gravimetric density of 10.4%. The adsorption energies of H_2_ molecules on Li_4_&B_36_ cluster are predicted in the range of 0.08-0.14 eV at the *w*B97*x* level of theory.

## Introduction

The hydrogen storage is an important issue not being solved up to now. Hydrogen can be adsorbed on a material through three different manners^1,2^. For chemisorption, the materials bind the dissociated hydrogen atoms with high binding energy of 2-4 eV, like metal hydrides and light complex hydrides^3,4^. The desorption occurs at high temperatures for the case of chemisorption. On the other hand, the materials, like nanostructured carbon^5,6^, attached hydrogen molecules weakly with a binding energy in the meV range, which was regarded as physisorption. The strength of third form of binding is in the 0.1–0.8 eV range, and is intermediate between physisorption and chemisorption. Metal-decorated carbon-based nanomaterials and even their derivates^7–13^, in which the metal atoms bind hydrogen in molecular form with intermediate binding energy, belong to the third form of binding. For example, the Li_12_C_60_ cluster was theoretically predicted to bind 60H_2_ at most, resulting in high gravimetric density^14^, and Yoon *et al*.^15^ have indicated that Ca can functionalize carbon fullerenes into high hydrogen storage capacity with a gravimetric density >8.4 wt%. In experiments, a lithium-doped fullerene with a Li:C_60_ mole ratio of 6:1 can reversibly desorb up to 5 wt % H_2_ with an onset temperature of ~270 °C^16^. Deuteration of Li_12_C_60_ was determined in experiment^17^, and the results indicated that up to 9.5 wt % deuterium (D_2_) are absorbed in Li_12_C_60_.

Highly stable clusters were generally designed in theory for the hydrogen storage^18–20^. For example, Ba Tai *et al*.^19^ have found that the B_6_Li_8_ cluster is a promising candidate for hydrogen storage media, which corresponds to a hydrogen uptake of 24% and adsorption energy of 0.099 eV estimated at the DFT level. Our previous work^21^ indicated that the CTi_7_^2+^cluster can bind 20 H_2_ molecules at most with adsorption energy of 0.24 eV, which can result into the gravimetric density of 19%. However, an ideal system is yet to be synthesized in experiment.

Boron fullerenes are also seen as efficient hydrogen storage media due to the light weight and capability to bind with metal adatoms. Many works reported the hydrogen storage of doped cage-like B_80_ with alkli-metal^22^, alkaline-earth metal^23,24^ and transitional metal^25^. In fact, B_80_ favors a core-shell (stuffed fullerene) structure in energy, not a cage-like configuration^26,27^. Therefore, the applications of B_80_ as hydrogen storage materials may be unrealistic. Recently, a combined experimental and theoretical study^28^ observed the first borospherenes (B_40_) with a cube-like cage structure, which brings the development of borospherene chemistry. Bai *et al*.^29^ have investigated the hydrogen storage of the lithium-decorated borospherene B_40_, and found the potential utilization of Li-B_40_ complexes as a novel nanomaterial for hydrogen storage. The Ti-decorated borospherene^30^ also theoretically studied as a promising hydrogen storage material, and the evaluated reversible storage capacity is 6.1 wt% for Ti-B_40_ complexes.

A high-symmetry C_6v_ quasi-planar structure with dual π aromaticity was predicted as the ground state of B_36_^31^. Nevertheless, by introducing four Li^+^ counterions into the B_36_^4−^ system, the neutral Saturn-like charge-transfer Li_4_&B_36_ complex with *D*_2h_ symmetry can be highly stabilized^32^. In the present work, we will pay attention to the hydrogen storage capability of Li_4_&B_36_ system.

## Results and Discussion

### Structures and bonding characters of Li_4_&B_36_ cluster

**Figure 1:**
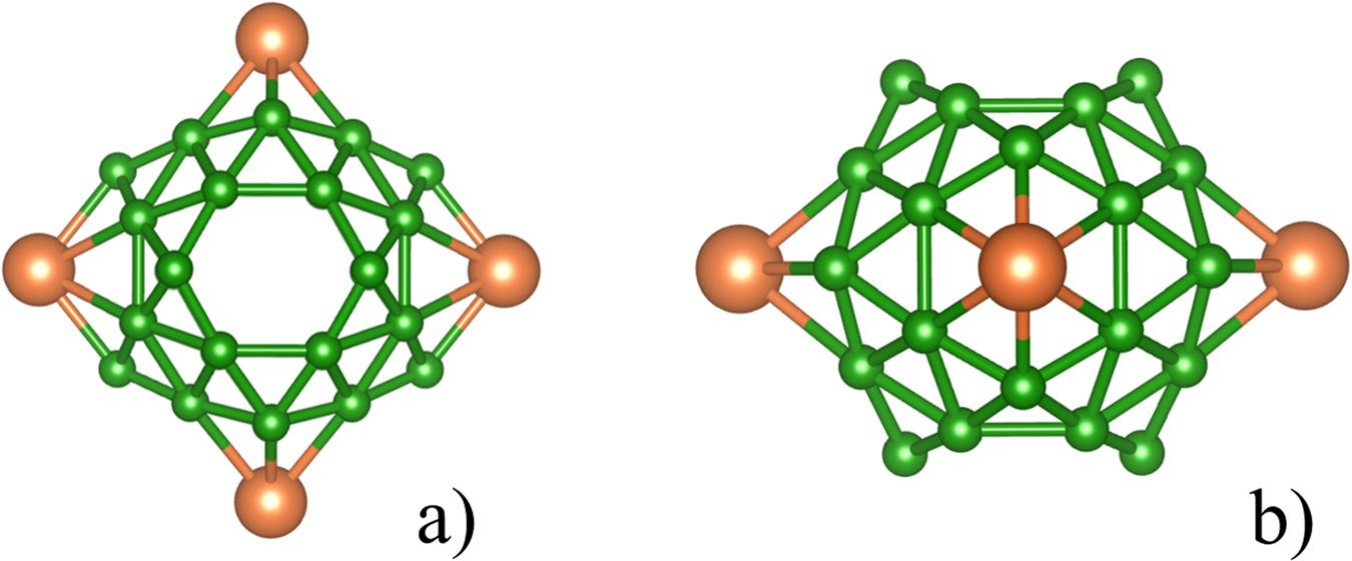
Relaxed structure of Li_4_&B_36_ cluster (*D*_2h_), a) side view, b) top view.

In previous work, the extensive structural search has found that the most stable structure of Li_4_&B_36_ cluster is one high-symmetry Saturn-like geometry^32^. As shown in Fig. 1, our calculations also obtained a cage structure with point group (PG) symmetry of *D*_2h_, this structure corresponds to a closed-shell electronic state (ES) (^1^A_g_). As Table 1 shows, the average Li-B and B-B bond lengths are 2.306 Å and 1.687 Å, respectively, predicted with *w*B97*x* functional^33^ in conjunction with 6-31 g (d, p) basis set, and the calculated bond lengths are excellent in agreement with previous results^32^. Natural population analysis (NPA)^34^ charge (shown in Table 1) indicate that each face-capping Li atom donating about one electron to the electron-deficient B_36_ core acts as electron donor, resulting into the (Li^+^)_4_B_36_^4−^ charge-transfer complex. From the electron configuration of Li atoms (*C*_Li_) in Li_4_&B_36_ cluster, one can find that the charge transfer from Li to B atoms result into the empty occupancy of 2 s valence shell. The sphere aromaticity of Li_4_&B_36_ is revealed by the huge negative nucleus-independent chemical shifts (NICS)^35^ of -44.6 ppm at the cage centers. The lowest vibrational frequency is 203 cm^−1^ at the *w*B97*x* level of theory, which is sufficiently large to meet a stability criterion suggested by Hoffmann *et al*.^36^. The high binding energy of 4.09 eV per Li atom also confirms the high stability of Li_4_&B_36_ cluster.

**Table 1:** The calculated structural parameters, the lowest frequency, NICS, NPA charge (*Q*_Li_) and electron configuration (*C*_Li_) of Li atoms and interaction energy (*E*_int_) of bare Li_4_&B_36_ cluster.

Species		*R*_Li-B_(Å)	*R*_B-B_(Å)	*ω*_L_ (cm^−1^)	NICS (0)	*Q*_Li_ (e)	*C*_Li_	*E*_int_ (eV)
Li_4_&B_36_	This work^a^	2.306	1.687	203	−44.6	0.86	1s^2^2s02p0^.1^	4.09
	Theory^b^	2.266	1.690	—	−42.8	0.83		3.27

^a^Our calculated values at the wB97x/6-31 G(d, p) level of theory.

^a^Predicted values at the PBE0/6-311 + G(d) level of theory from ref ^32^.

**Figure 2:**
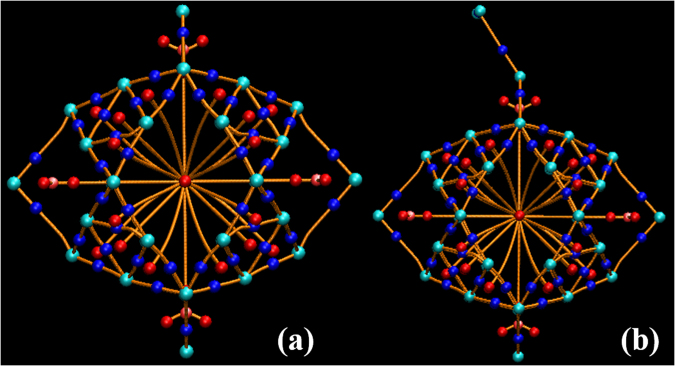
Molecular graph of Li_4_&B_36_ (**a**) and Li_4_&B_36_-H_2_ (**b**) complexes. The colour scheme identifying critical points is as follows: cyan ball for attractors, blue ball for bond critical points (BCP), red ball for ring critical points (RCP).

The bonding nature of Li_4_&B_36_ cluster will be revealed with QTAIM method^37^, the molecular graphs and corresponding topological parameters are given in Fig. 2 and Table 2, respectively. Different (3, -1) bond critical points (BCPs) relative to B-B bonds and the bond critical points (BCPs) between Li atoms and two neighboring B atoms are found. For the traditional topological criterion, the covalent interaction corresponds to a negative Laplacian of electron density (∇^2^ρ(r) < 0) at the BCP. Another property, the total energy density *H*(r) (defined as the sum of local kinetic energy density *G*(r) and local potential energy density *V*(r)) proposed by Cremer and Krala^38^ was proven to be very appropriate to characterize the degree of covalency of a bond. The negative *H*(r) is the indicator of a covalent bond. As Table 2 shows, all bond critical points relative to Li-B bonds correspond to positive Laplacian of electron density ∇^2^ρ(r) and *H*(r) value. This indicates that the Li-B bonds show typical closed-shell character corresponding to ionic bonds. On the other hand, the covalent bond nature of B-B bonds is revealed by their large electron density ρ(r), negative ∇^2^ρ(r) and *H*(r) values. This result is in excellent agreement with fuzzy bond order (FBO)^39^ analyses, which predicts the bond order between Li and B atoms to be 0.23, suggesting the weak ionic bond nature of Li-B bonds. And the high fuzzy bond order (FBO) of B-B bonds reveal the strong covalent interaction between the bonding B atoms. The topological parameters of electron density shown in Table 2 indicate that all Li-B and B-B chemical bonds in Li_4_&B_36_ show typical ionic and covalent natures, respectively, which is also supported by the electron localization function (ELF)^40,41^ shown in Table 2.

**Table 2:** Topological parameters of isolated H_2_ molecule and Li_4_&B_36_ cluster and H_2_-adsorbed Li_4_&B_36_-H_2_ complex.

Species	BCP	*ρ*	∇^2^*ρ*	*H*(r)	FBO	ELF
H_2_	H-H	0.270	−1.219	−0.305	1.00	1.00
Li_4_&B_36_	B-Li	0.026	0.113	0.001	0.23	0.05
	B-B	0.127 (0.151)^a^	−0.090 (−0.271)	−0.073 (−0.115)	0.56 (0.87)	0.69 (0.87)
Li_4_&B_36_-H_2_	B-Li	0.026	0.113	0.001	0.23	0.05
	B-B	0.127 (0.151)	−0.090 (−0.271)	−0.073 (−0.115)	0.56 (0.87)	0.69 (0.87)
	Li-H_2_	0.008	0.043	0.002	0.21	0.01
	H-H	0.267	−1.190	−0.299	0.76	1.00

^a^parameters for the shortest B-B bonds were shown in the parentheses.

### H_2_ adsorption on Li_4_&B_36_ cluster

In this section, we will pay attention to the hydrogen storage stability of Li_4_&B_36_ cluster. The bond length of free H_2_ molecule is predicted as 0.744 Å at the *w*B97*x*/6-31 g (d, p) level of theory, and is in agreement with the experimental value of 0.741 Å^42^. The free and adsorbed H_2_ molecules in Li_4_&B_36_ show almost the same topological parameter as shown in Table 2. This indicates that the hydrogen is attached by the cluster in molecule form. The geometry of Li_4_&B_36_ cluster is maintained after H_2_ being attached comparing with isolated one. The molecular graph of Li_4_&B_36_-H_2_ is also shown in Fig. 2, from which one can find that one bond critical point between Li atom and H_2_ molecule is localized. The corresponding electron density, and other topological parameters (∇^2^ρ, H(r)) of Li-H_2_ BCP suggest that the Li-H_2_ interaction shows weak noncovalent characteristic. In addition, we note that the topological parameter of bond critical points relative to Li-B and B-B bonds in H_2_-adsorbed species are almost the same to those of isolated Li_4_&B_36_ cluster. This further demonstrates that the structure of host cluster was not distorted after H_2_ being attached.

**Figure 3:**
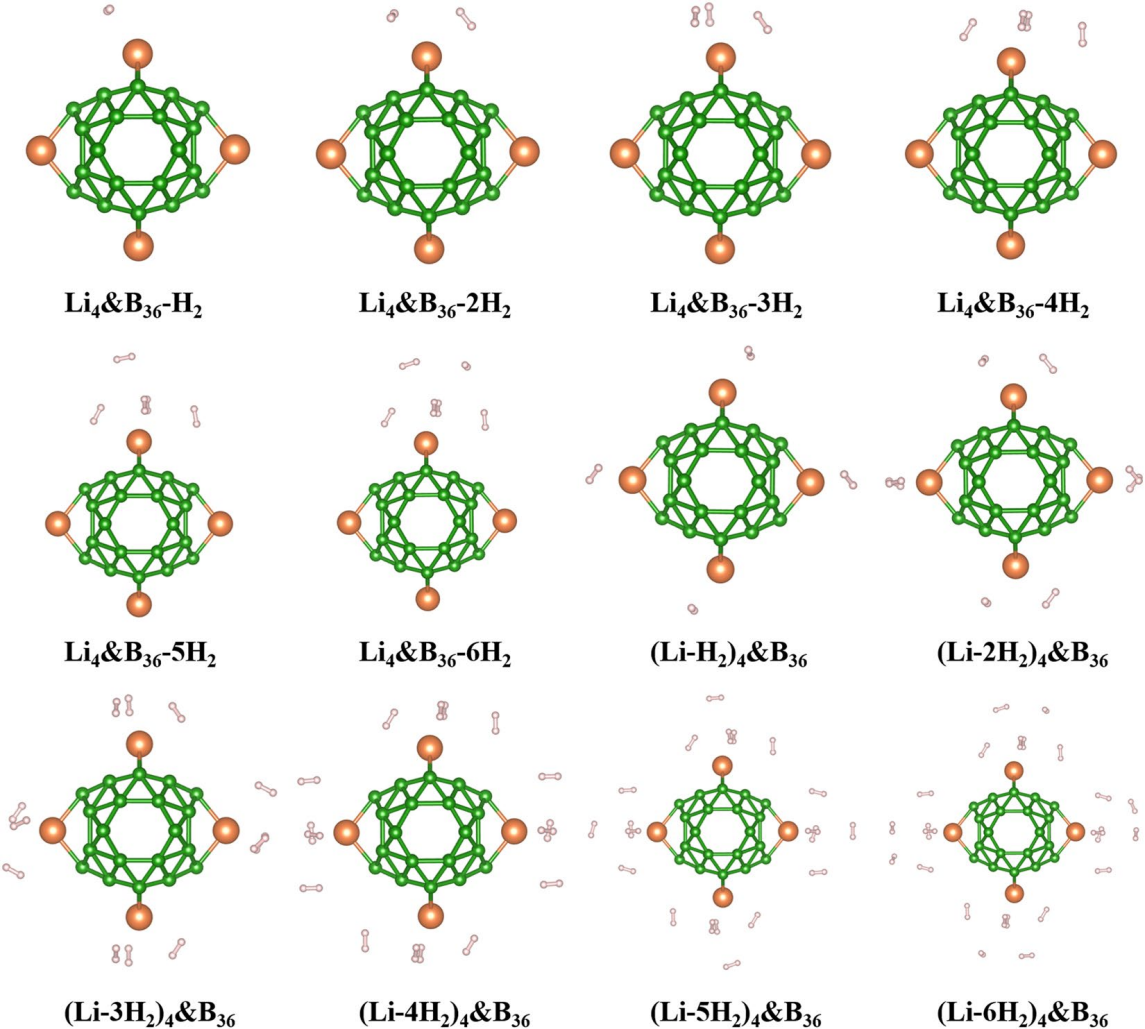
Optimized structure of H_2_ molecules adsorbed Li_4_&B_36_ cluster.

The nH_2_-adsorbed configurations were extensively optimized to probe into the hydrogen storage stability. Vibrational frequency calculations confirmed that all the relaxed nH_2_-adsorbed structures are to be local stable, and the Cartesian coordinates of these species are listed in Table S1 of supporting information. The relaxed configurations are depicted in Fig. 3. Our calculations indicate that each Li atom in Li_4_&B_36_ cluster can attach six H_2_ molecules at most. The average Li-B and B-B bond lengths of nH_2_-adsorbed species are collected in Table 3, from which one can see that the B-B bond lengths in adsorbed species are not changed relative to the isolated cluster. On the other hand, the Li-B bonds are gradually elongated as the numbers of adsorbed H_2_ molecules increase due to the increased interaction between Li atoms and H_2_ molecules. The largest elongation of 0.025 Å is found for (Li-6H_2_)_4_&B_36_ species. Therefore, the H_2_ adsorptions do not result into the high structure distortion of Li_4_&B_36_ cluster. The bond lengths of adsorbed H_2_ molecules are elongated by only 0.7% relative to the isolate H_2_ molecule (0.774 Å). It is obvious that the H-H bonds are not broken after being adsorbed on Li_4_&B_36_ cluster. The Li–H_2_ distances are in a rather wide range from 2.207 Å to 3.080 Å as shown in Table 3. It is observable that there is an abrupt increase in the Li–H_2_ bond lengths from Li_4_&B_36_-4H_2_ to Li_4_&B_36_-5H_2_, so that the first four H_2_ molecules are closer to the Li site than the next one. By comparing Li_4_&B_36_-4H_2_ and (Li-H_2_)_4_&B_36_, one can find that the Li-B bonds are more elongated in Li_4_&B_36_-4H_2_ (4 H_2_ coadsorption on one Li atom) than those in (Li-H_2_)_4_&B_36_ which corresponds to uniform adsorption on 4 Li atoms. Moreover, the adsorption energy of (Li-H_2_)_4_&B_36_ is significantly larger than that of Li_4_&B_36_-4H_2_. This indicates that the hydrogen molecules tend to uniformly be attached by 4 Li atoms.

**Table 3:** Calculated structural parameters, NPA charge (*Q*_Li_) and electron configuration (*C*_Li_) of Li atom, adsorption energy (*E*_ads_) and consecutive adsorption energy (*E*_r_) of H_2_-adsorbed Li_4_&B_36_ species.

Species	*R*_Li-B_(Å)	*R*_B-B_(Å)	*R*_H-H_(Å)	*R*_Li-H_(Å)	*Q*_Li_(e)	*C*_Li_	*E*_ads_ (eV)	*E*_r_ (eV)
Li_4_&B_36_-H_2_	2.306	1.687	0.748	2.207	0.74	1s^2^2 s^0^2p^0.2^0	0.14	0.14
Li_4_&B_36_-2H_2_	2.309	1.687	0.749	2.257	0.60	1s^2^2s^0^2p^0.3^0	0.13	0.12
Li_4_&B_36_-3H_2_	2.311	1.687	0.749	2.407	0.54	1s^2^2s^0^2p^0.36^	0.11	0.07
Li_4_&B_36_-4H_2_	2.314	1.687	0.748	2.645	0.52	1s^2^2s^0^2p^0.37^	0.10	0.07
Li_4_&B_36_-5H_2_	2.314	1.687	0.748	2.914	0.52	1s^2^2s02p^0.37^	0.09	0.04
Li_4_&B_36_-6H_2_	2.315	1.687	0.748	3.080	0.53	1s^2^2s^0^2p^0.37^	0.08	0.04
(Li-H_2_)_4_&B_36_	2.305	1.687	0.749	2.218	0.73	1s^2^2s^0^2p^0.20^	0.13	—
(Li-2H_2_)_4_&B_36_	2.309	1.687	0.749	2.258	0.60	1s^2^2s^0^2p^0.30^	0.13	—
(Li-3H_2_)_4_&B_36_	2.326	1.687	0.749	2.416	0.54	1s^2^2s^0^2p^0.36^	0.11	—
(Li-4H_2_)_4_&B_36_	2.327	1.687	0.748	2.674	0.53	1s^2^2s^0^2p^0.37^	0.10	—
(Li-5H_2_)_4_&B_36_	2.330	1.687	0.748	2.935	0.53	1s^2^2s^0^2p^0.37^	0.08	—
(Li-6H_2_)_4_&B_36_	2.331	1.687	0.748	3.011	0.53	1s^2^2s^0^2p^0.36^	0.08	

We calculated consecutive adsorption energy (*E*_r_) as the energy gained by successive additions of H_2_ molecules to evaluate the reversibility for storage of H_2_ molecules. The average adsorption energy (*E*_ads_) was calculated to evaluate the adsorption capability of the Li_4_&B_36_ cluster. They are defined as follows:

1 $${E}_{r}=[E(L{i}_{4}\& {B}_{36}-(n-1){H}_{2})+E({H}_{2})-E(L{i}_{4}\& {B}_{36}-n{H}_{2})$$

2 $${E}_{ads}=[E(L{i}_{4}\& {B}_{36})+nE({H}_{2})-E(L{i}_{4}\& {B}_{36}-n{H}_{2})]/n$$

where, $E(L{i}_{4}\& {B}_{36})$, $E({H}_{2})$, $E(L{i}_{4}\& {B}_{36}-n{H}_{2})$,$E(L{i}_{4}\& {B}_{36}-(n-1){H}_{2})$ are the total energy of Li_4_&B_36_ cluster, H_2_, Li_4_&B_36_-nH_2_, and Li_4_&B_36_-(n-1)H_2_, respectively. The *E*_r_ is an important index for testing the continuous hydrogen adsorption capacity of nanomaterials. The adsorption of H_2_ is difficult if the *E*_r_ is negative^25^, and the positive *E*_r_ means the spontaneous adsorption can occur between the hydrogen molecule and the Li_4_&B_36_ structure. From Table 3, one can be found that the *E*_r_ for the sixth H_2_ adsorbed by one Li atom is 0.04 eV. Therefore, we can conclude that each Li atom can at most attach six H_2_ molecules in stable state. We note that a Li atom in Li-decorated B_40_ also attach six H_2_ molecules at most in previous work^29^. The adsorption of 24 H_2_ on the present system (Li_4_&B_36_) corresponds to a gravimetric density of 10.4%. It is obvious that the gravimetric density exceeds the 5.5 wt% at 2017 specified by the US department of energy (DOE). It can be seen from Table 3 that the *E*_ads_ are in the range of 0.08eV–0.14 eV calculated at the *w*B97*x*/6-311++g(2d, 2p) level of theory. These values are very close to the average bonding energy for lithium coated fullerene Li_12_C_60_^14^, aromatic B_6_Li_8_^19^ complex, and lithium-decorated borospherene Li_6_B_40_^29^.

It can be seen from Table 3 that the Li atoms in all nH_2_-adsorbed species act as electron donor, and the charge transfer is decreased as the numbers of adsorbed H_2_ molecules increase. In addition, the 2s→2p electron promotion occurs in the Li atoms after H_2_ being adsorbed, which results into the non-empty occupancy in 2p orbital (0.20–0.37e) of Li atoms.

**Figure 4:**
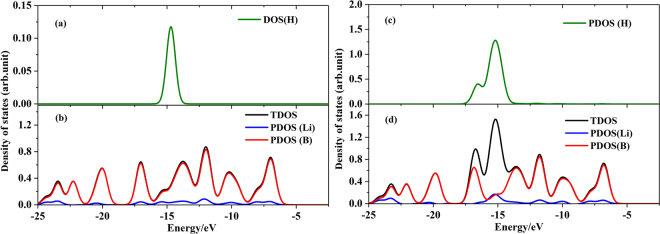
Partial density of states (PDOS) of isolated H_2_ (**a**) and Li_4_&B_36_ (**b**), adsorbed H_2_ (**c**) and Li_4_&B_36_ (**d**) in Li_4_&B_36_-20H_2_ complex.

Partial density of states (PDOS) of free H_2_ and Li_4_&B_36_ and 20H_2_-adsorbed (Li-5H_2_)_4_&B_36_ complexes are analyzed to understand the bonding characteristics of hydrogen adsorbed systems. The PDOS plots are depicted in Fig. 4. For Li_4_&B_36_ cluster, there exists very weak orbital overlaps between B and Li atoms, which is in agreement with the ionic characters revealed by aforementioned QTAIM analyses. Comparing to the free Li_4_&B_36_ cluster, there exists new peak around −15eV in the 20H_2_-adsorbed complex, stemming from the 2p electron of Li atoms due to the 2 s→2p electron promotion. Additionally, the new peak can participate into the orbital overlaps with H_2_ molecules. This results into the physical interaction between H_2_ molecules and Li_4_&B_36_ cluster.

## Conclusion

The Saturn-like charge-transfer complexes Li_4_&B_36_ cluster were investigated as a candidate for hydrogen storage with density functional theory (DFT) methods. The bonding nature in bare and H_2_-adsorbed Li_4_&B_36_ clusters was revealed with QTAIM analyses. Our results suggest that each face-capping Li atom donates about one electron to the electron-deficient B_36_ core, resulting into the (Li^+^)_4_B_36_^4−^ charge-transfer complex. The ionic characters of Li-B bonds and covalent characters of B-B bonds are understood for bare Li_4_&B_36_ cluster. Each Li atom in Li_4_&B_36_ cluster can at most attach five H_2_ molecules, which results into the gravimetric density of 10.4%, exceeding the 5.5 wt% at 2017 specified by the US department of energy (DOE). The structure distortion of Li_4_&B_36_ cluster was not occurred after the H_2_ molecules were attached. The adsorption energies of H_2_ molecules on Li_4_&B_36_ cluster are in the range of 0.08-0.14 eV at the *w*B97*x*/6-311++g(2d, 2p) level of theory. These values are very close to the average bonding energy for lithium coated fullerene Li_12_C_60_, aromatic B_6_Li_8_ complex and lithium-decorated borospherene Li_6_B_40_. Our study indicates that the Li_4_&B_36_ cluster may be appropriate material for hydrogen storage, but also need further confirmation in experiment.

## Method

All the calculations were carried out with G09 package^43^. The molecular structures of bare and nH_2_-adsorbed Li_4_&B_36_ species were fully relaxed without any symmetry constrains using *w*B97*x* functional^33^. This functional has considered the long-rang corrections, and is proved to be reliable methods to predict non-covalent interactions. The classical extended basis set 6-31 g (d, p) was utilized in the geometry optimization. By adding H_2_ molecules around the Li atoms to construct the starting adsorption configurations of Li_4_&B_36_-nH_2_ (n=1–20) which were then full relaxed at the *w*B97*x*/6-31 G(d, p) level of theory. The harmonic vibrational frequency calculations were carried out at the same level of theory to guarantee that the optimized structures correspond to local minima on the potential energy surface. The larger basis set, 6-311++g(2d, 2p), was employed in the single-point energy calculations to obtain the more reasonable adsorption energy.

To understand the bonding characters of the studied systems, the quantum theory of atoms in molecules (QTAIM)^37^ and natural population analyses (NPA)^34^ were performed with MULTIWFN program^44^.

## Electronic supplementary material

Supporting information
