# Supplementary material for: Hydrogen storage of Li4&B36 cluster
Source: Sci Rep. 2018 Jan 31;8:1940. doi: 10.1038/s41598-018-20452-8 (PMC5792484; doi:10.1038/s41598-018-20452-8)
Supplement: Supplementary file 1 — Supporting information [file 41598_2018_20452_MOESM1_ESM.pdf]

Supporting information

Hydrogen storage of Li_4&B_{36} cluster

Jiguang Du[a*], Xiyuan Sun[b], Li Zhang[c], Chuanyu Zhang[d], Gang Jiang[c]
[a]College of Physical Science and Technology, Sichuan University, Chengdu 610064, China
[b]College of Science, Sichuan Agricultural University, Ya'an 625014, China
[c]Institute of Atomic and Molecular Physics, Sichuan University, Chengdu 610065, China
[d] Department of Physics, Chengdu University of Technology, Chengdu 610059, China

Table S1. Cartesian coordinate of nH_2-adsorbed Li_4&B_{36} species.

Li_4&B_{36}-H_2:

B	-2.24692200	-1.19640600	-1.29679600
B	0.31191600	-2.51115400	1.78363800
B	1.21828100	2.42431300	0.61363900
B	-0.91125900	1.42569300	2.17147600
B	2.42203500	-0.60873000	1.27400900
B	1.14727400	2.30596300	-1.16144800
B	2.31074200	-0.79505900	-1.50722100
B	-1.61678800	2.06245600	-1.03405600
B	0.17661300	-2.73746600	-1.58846600
B	2.26124000	1.15858700	1.16235700
B	-1.20432500	-2.46289100	-0.74799900
B	-0.29811300	2.47348100	-1.91826100
B	2.48605200	1.89884800	-0.29508900
B	0.85918500	1.58171000	2.08994000
B	-2.40762200	0.57066800	-1.40809600
B	-1.54608000	2.18168300	0.74126900
B	-1.09400400	1.11897900	-2.39669800
B	-1.55737400	0.00943900	2.63508800
B	2.79361200	-1.47282900	-0.08206700

* Corresponding author:
E-mail: dujg@scu.edu.cn

B	1.63064000	-2.09998200	0.89953400
B	0.92548200	-1.46377200	-2.30630600
B	1.57074600	-0.04753200	-2.76944700
B	-2.77981100	1.43567000	-0.05253000
B	-0.16267800	2.69934000	1.45421900
B	-0.84518800	-1.61931000	-2.22449100
B	-1.76839900	-0.34216100	-2.61578100
B	-0.66228500	-1.31363900	2.34498000
B	1.56036500	-2.21984200	-0.87585000
B	-1.13329500	-2.34421100	1.02680300
B	2.14911900	0.97206100	-1.61801000
B	1.78219500	0.30410800	2.48132200
B	-2.29670700	0.75703000	1.37237900
B	0.67606400	1.27630000	-2.47941900
B	1.10842400	-1.15686100	2.26267700
B	-2.13513600	-1.00996600	1.48360400
B	-2.47200600	-1.93722500	0.16061800
Li	-0.34835500	3.93125300	-0.31964600
Li	0.36722000	-3.97090000	0.18577200
Li	3.98569200	0.33459200	-0.25128400
Li	-3.97119600	-0.37242400	0.11662300
H	-1.64096600	5.70110800	-0.03936800
H	-1.74248200	5.57719000	-0.77051100

Li_4&B_{36}-2H_2:

B	-2.24110600	-1.28633800	-1.29510200
B	0.35657800	-2.51379300	1.78950900
B	1.11246200	2.44123600	0.60293000
B	-0.98452600	1.38528000	2.16212000
B	2.40656400	-0.54893200	1.27231300
B	1.04582300	2.31490600	-1.17034300

B	2.30346300	-0.74714500	-1.50666200
B	-1.70814300	1.99103200	-1.04284000
B	0.22910400	-2.75407400	-1.58161900
B	2.19372200	1.21069100	1.15643700
B	-1.15954900	-2.51819600	-0.74205100
B	-0.40254600	2.43860300	-1.92885400
B	2.39775100	1.95520100	-0.30296000
B	0.77863300	1.59426200	2.08151600
B	-2.45364000	0.47518300	-1.41107700
B	-1.64262000	2.11918900	0.73064800
B	-1.15805600	1.05936500	-2.40205500
B	-1.58976100	-0.04747900	2.63174200
B	2.80743600	-1.40770400	-0.07972800
B	1.66079900	-2.06336600	0.90319400
B	0.93792500	-1.45960600	-2.30253400
B	1.54388400	-0.02725000	-2.77239300
B	-2.85264400	1.33314200	-0.05973000
B	-0.27538300	2.68021400	1.44221000
B	-0.82555000	-1.66845600	-2.21970200
B	-1.78956900	-0.42207100	-2.61671400
B	-0.65312500	-1.34389800	2.34479900
B	1.59456200	-2.19052100	-0.86996100
B	-1.09311000	-2.39331700	1.03131000
B	2.08730600	1.01191300	-1.62171200
B	1.74262200	0.34752000	2.47850800
B	-2.34912300	0.67316500	1.36579800
B	0.60700300	1.26932300	-2.48594600
B	1.11164500	-1.13453500	2.26412000
B	-2.13319400	-1.08705200	1.48140400
B	-2.44324600	-2.02934900	0.16376200

Li	-0.49515800	3.90924500	-0.33724600
Li	0.46287000	-3.97986500	0.19617000
Li	3.94956700	0.43524600	-0.25509700
Li	-3.99713600	-0.50988900	0.11678200
H	-2.12591900	5.37515400	0.07089500
H	-2.03225200	5.51227900	-0.65896800
H	1.21125500	5.35801400	-0.50825800
H	0.68323400	5.88877700	-0.50550700

Li_4&B_{36}-3H_2:

B	-2.21752900	-1.10107000	-1.30873200
B	0.24304100	-2.59760400	1.76949100
B	1.23814700	2.37716800	0.86787300
B	-0.94424400	1.33128700	2.31840000
B	2.38899700	-0.70026800	1.41050200
B	1.21161300	2.34559200	-0.91224700
B	2.34956300	-0.74877300	-1.38011200
B	-1.55797800	2.13374800	-0.86966900
B	0.19506300	-2.65693900	-1.61091400
B	2.24873200	1.07136900	1.37982600
B	-1.20483200	-2.40588100	-0.79457900
B	-0.21001700	2.56801000	-1.69442000
B	2.52284300	1.88195200	-0.03229900
B	0.82905700	1.46730000	2.28972800
B	-2.35381000	0.67087800	-1.33623900
B	-1.53005500	2.16114300	0.91032300
B	-1.00944700	1.25117900	-2.26324300
B	-1.61903600	-0.09801800	2.69574500
B	2.78503800	-1.50054000	0.02285200
B	1.58938900	-2.16125800	0.94132600
B	0.97797200	-1.36086800	-2.24721100

B	1.65172300	0.06771400	-2.62242400
B	-2.75232400	1.47314600	0.04968900
B	-0.16157100	2.62751100	1.68112000
B	-0.79631000	-1.49651400	-2.21851300
B	-1.69330100	-0.18967900	-2.57074600
B	-0.73206200	-1.41691000	2.36441300
B	1.56466700	-2.19253500	-0.83775300
B	-1.17882500	-2.37380500	0.98395500
B	2.20944500	1.02346500	-1.40789200
B	1.72627000	0.15989100	2.64305700
B	-2.31531200	0.71984500	1.45188400
B	0.76369800	1.38687700	-2.28928700
B	1.04271400	-1.28001700	2.33659300
B	-2.17876800	-1.05299000	1.48224700
B	-2.48973500	-1.90803800	0.10516600
Li	-0.29711100	3.98745600	-0.03337700
Li	0.32235600	-3.97878600	0.10272900
Li	4.00154700	0.29619000	-0.02575200
Li	-3.96914600	-0.32538100	0.09981200
H	-1.47174800	5.36212400	1.36838400
H	-1.48754600	5.87280600	0.82101400
H	-0.91357500	5.37841600	-1.98516200
H	-0.99968200	6.02243500	-1.61314600
H	1.55763100	5.31240900	0.16806800
H	1.09503500	5.86277800	-0.04015200

Li_4&B_{36}-4H_2:

B	-1.39803600	-2.17950300	1.36244200
B	-2.80676800	0.33113100	-1.71821400
B	2.21014500	1.11658000	-0.84947000
B	1.06344900	-1.02294300	-2.28331300

B	-0.81780300	2.39646600	-1.37955100
B	2.18941600	1.10094200	0.93019000
B	-0.85123700	2.37080500	1.41131900
B	1.85764400	-1.65506300	0.90036300
B	-2.84696600	0.29962200	1.66333800
B	0.94737600	2.18190600	-1.35920200
B	-2.66074800	-1.11247900	0.85100000
B	2.35653900	-0.32603000	1.71658000
B	1.77553400	2.42672600	0.04735800
B	1.27710700	0.74235400	-2.26558700
B	0.36797200	-2.38950100	1.37925700
B	1.88083600	-1.64033000	-0.88040300
B	1.00859900	-1.06581700	2.29539700
B	-0.39413800	-1.63901500	-2.65012700
B	-1.59319000	2.83244600	0.01091700
B	-2.30807000	1.66153300	-0.89858600
B	-1.51306600	1.02881400	2.28633600
B	-0.05634800	1.64468300	2.65247200
B	1.14691200	-2.82799300	-0.00946400
B	2.39734500	-0.29488800	-1.65868500
B	-1.72625400	-0.73808700	2.26761000
B	-0.45721300	-1.68886300	2.61586200
B	-1.67186100	-0.69520400	-2.31482200
B	-2.32932200	1.64510100	0.88111100
B	-2.63998100	-1.09613600	-0.92805300
B	0.91365600	2.15601300	1.42873300
B	0.00672400	1.69379600	-2.61475000
B	0.40126900	-2.36316200	-1.40779100
B	1.22206000	0.69991100	2.31588100
B	-1.45813800	1.07144200	-2.29502000

B	-1.36414600	-2.15275300	-1.42820600
B	-2.22386200	-2.42185600	-0.04566300
Li	3.77564200	-0.47623900	0.04335000
Li	-4.17268200	0.47720300	-0.04258600
Li	0.25449700	3.97244500	0.04360400
Li	-0.70329200	-3.96723200	-0.04296200
H	5.36209000	-0.17318900	-1.72873100
H	5.91883000	-0.18370600	-1.22832000
H	5.86791800	-0.00795500	1.22289700
H	5.32657100	-0.01460400	1.74022100
H	5.26669600	-2.35591100	0.14776200
H	4.59323000	-2.67164100	0.06496400
H	4.97065000	2.52049800	-0.13873700
H	5.70434700	2.41472700	-0.23057500

Li_4&B_{36}-5H_2:

B	-1.21335000	-2.29687200	1.37927800
B	-2.92027600	0.02713300	-1.69671900
B	1.98475000	1.37093000	-0.87512900
B	1.07290200	-0.88931200	-2.29246000
B	-1.17135600	2.30297100	-1.38241800
B	1.98228600	1.36126400	0.90479900
B	-1.17767300	2.28503300	1.40824200
B	1.95868600	-1.41467200	0.88654100
B	-2.92817400	0.00578400	1.68538300
B	0.60672900	2.28684900	-1.37670100
B	-2.59203300	-1.38025800	0.87595200
B	2.31421900	-0.03510200	1.69362400
B	1.41540300	2.62876100	0.02120200
B	1.08727100	0.88923500	-2.28123900
B	0.56446300	-2.30855700	1.38150200

B	1.96406000	-1.40440400	-0.89370300
B	1.06249900	-0.91836500	2.28813000
B	-0.31058100	-1.66492900	-2.64386200
B	-1.97869600	2.65548500	0.01340800
B	-2.56638800	1.40809700	-0.88577500
B	-1.67911900	0.88208300	2.29419300
B	-0.29647700	1.65684100	2.64454900
B	1.37507500	-2.66349500	-0.01277800
B	2.32202000	-0.01394400	-1.68109200
B	-1.69315600	-0.89782300	2.28310500
B	-0.32279800	-1.69859900	2.62324900
B	-1.68260000	-0.86865600	-2.30008000
B	-2.57093000	1.39710700	0.89398900
B	-2.58808200	-1.36919800	-0.90332000
B	0.59996400	2.26872700	1.41052400
B	-0.28427000	1.69079600	-2.62242100
B	0.57090900	-2.29091800	-1.40613900
B	1.07667900	0.86017900	2.29972100
B	-1.66830300	0.91132900	-2.28828400
B	-1.20671100	-2.27891200	-1.41133500
B	-2.01971500	-2.63682100	-0.02027100
Li	3.73469300	-0.03828600	0.00905200
Li	-4.27727300	0.02698000	-0.00858700
Li	-0.27153300	3.99172300	0.02542800
Li	-0.33447800	-3.99954200	-0.02499500
H	5.27107300	0.24406100	-1.77826900
H	5.83496600	0.21150800	-1.28636400
H	5.81288400	0.32980300	1.24585600
H	5.25896800	0.34150200	1.75003200
H	5.39394300	-1.93353900	0.09137500

H	4.73287500	-2.27795500	0.02310800
H	4.63628800	2.78625600	-0.06444700
H	5.36786300	2.65803500	-0.14694700
H	7.92536900	-0.43514200	-0.01099200
H	7.77538400	-1.15598400	0.10561500

Li_4&B_{36}-6H_2:

B	-1.48739900	-2.21770400	1.35544300
B	-2.97574700	0.26872300	-1.70700200
B	2.02084800	1.18420000	-0.85253000
B	0.92817600	-0.97799300	-2.29365100
B	-1.04182000	2.38538100	-1.36555900
B	2.00866500	1.15975400	0.92681100
B	-1.06356400	2.34428800	1.42562400
B	1.75071000	-1.60384500	0.88387500
B	-3.00222400	0.21931800	1.67457700
B	0.72845300	2.21814300	-1.35239100
B	-2.78094600	-1.18311700	0.85432000
B	2.21762100	-0.26618200	1.70498300
B	1.55501700	2.47790900	0.05235700
B	1.09364100	0.79255000	-2.26720000
B	0.28333000	-2.38059200	1.36503500
B	1.76629500	-1.57960900	-0.89645600
B	0.89230300	-1.04490600	2.28546700
B	-0.51418600	-1.63066000	-2.65804300
B	-1.82287700	2.79296400	0.03037400
B	-2.50974100	1.60780100	-0.88231200
B	-1.68625300	0.98103500	2.29654400
B	-0.24483000	1.63333400	2.66013300
B	1.06804100	-2.79115800	-0.02896900
B	2.24403900	-0.21712100	-1.66972200

B	-1.85122800	-0.79062600	2.26929400
B	-0.55552800	-1.70826800	2.60816400
B	-1.81567100	-0.72357700	-2.31310000
B	-2.52421100	1.58237100	0.89734700
B	-2.76721400	-1.15733600	-0.92470400
B	0.70629100	2.17710800	1.43554400
B	-0.20341400	1.71091600	-2.60710400
B	0.30506700	-2.33983500	-1.42244200
B	1.05760700	0.72558100	2.31372300
B	-1.64988000	1.04801000	-2.28512900
B	-1.46521700	-2.17657800	-1.43506300
B	-2.31218500	-2.47560500	-0.05064800
Li	3.63937500	-0.37078800	0.02587400
Li	-4.33795700	0.36981300	-0.02532800
Li	-0.00596500	3.98105200	0.06274100
Li	-0.75147500	-3.97835200	-0.06048800
H	5.23453400	-0.07736300	-1.73908900
H	5.78836300	-0.09782200	-1.23491200
H	5.74685200	0.05697700	1.19665000
H	5.20598400	0.07076600	1.71517100
H	5.14549100	-2.26242600	0.10243400
H	4.47239700	-2.58445400	0.04024400
H	4.72094500	2.59287900	-0.13974700
H	5.45423800	2.47609500	-0.22463700
H	7.78265400	-1.21721700	0.09719600
H	7.49594300	-1.89925800	0.18938200
H	7.83210200	1.30188000	-0.43973400
H	7.72871100	1.41139800	0.29068800

$(Li\text{-}H_2)_4\&B_{36}$:

B	-2.18043600	-1.24506400	-1.43890800

B	0.41633400	-2.53134900	1.62407700
B	1.04958700	2.49253700	0.65510700
B	-1.03587800	1.31124900	2.14379300
B	2.41674000	-0.49134300	1.21255900
B	1.00492900	2.43683100	-1.12292500
B	2.35027800	-0.57504900	-1.57287900
B	-1.74201300	2.03195300	-1.04232900
B	0.33176500	-2.63062200	-1.75679300
B	2.15772700	1.26674000	1.16934900
B	-1.07235200	-2.46939100	-0.92535900
B	-0.43888300	2.55154700	-1.89211200
B	2.35560700	2.07539700	-0.25526400
B	0.72200400	1.57121500	2.09160600
B	-2.44237600	0.51350000	-1.48444700
B	-1.69725000	2.08541100	0.73544700
B	-1.15149400	1.17444500	-2.43349100
B	-1.60552700	-0.15577100	2.54481300
B	2.85451500	-1.27998800	-0.16838400
B	1.71881900	-2.01068400	0.77259200
B	1.01408200	-1.28920500	-2.41495500
B	1.58292900	0.17768600	-2.81527900
B	-2.87776300	1.30150800	-0.10123300
B	-0.35474500	2.65409100	1.48772800
B	-0.74575600	-1.54957700	-2.36284400
B	-1.73710400	-0.31276500	-2.71692400
B	-0.63097300	-1.41358300	2.21597200
B	1.67473900	-2.06303500	-1.00622700
B	-1.02772500	-2.41709800	0.85393200
B	2.08783200	1.18313300	-1.61761700
B	1.71430800	0.33485700	2.44679300

B	-2.37206300	0.59634000	1.30203900
B	0.60849800	1.43465800	-2.48555600
B	1.12847100	-1.15345900	2.16421900
B	-2.10887800	-1.16085900	1.34667800
B	-2.37788400	-2.05412100	-0.01411100
Li	-0.59834200	3.94136700	-0.23641900
Li	0.57587200	-3.91732900	-0.03072800
Li	3.94411700	0.59559100	-0.25498100
Li	-3.96603300	-0.57611200	-0.01997100
H	5.42923300	1.23258000	1.23947500
H	5.88980300	0.79684000	0.84155300
H	-5.51953600	-0.23928800	1.49611400
H	-5.83219800	-0.85257400	1.20196400
H	0.36648200	5.72427000	-1.05986600
H	0.23577200	6.01934000	-0.38435000
H	-0.41649500	-5.81939600	0.48410000
H	-0.31100400	-5.94076900	-0.24693400

$(Li\text{-}2H_2)_4\&B_{36}$:

B	-2.23942700	-1.28647000	-1.29570400
B	0.35702300	-2.51080900	1.79114500
B	1.10957100	2.44463200	0.60356700
B	-0.98751900	1.38750500	2.16181500
B	2.40601600	-0.54440200	1.27491000
B	1.04433600	2.31754300	-1.16974000
B	2.30484800	-0.74357200	-1.50404700
B	-1.70940200	1.99152800	-1.04417200
B	0.23212200	-2.75223700	-1.57996200
B	2.19108000	1.21489700	1.15801700
B	-1.15715100	-2.51706400	-0.74138200
B	-0.40348900	2.43984000	-1.92934200

B	2.39595700	1.95927200	-0.30123000
B	0.77550100	1.59785100	2.08235400
B	-2.45345600	0.47488900	-1.41240400
B	-1.64517100	2.12039500	0.72948900
B	-1.15770000	1.05989200	-2.40257000
B	-1.59179300	-0.04575600	2.63121100
B	2.80842700	-1.40342600	-0.07664000
B	1.66150000	-2.05960400	0.90567600
B	0.94056400	-1.45748300	-2.30064800
B	1.54560200	-0.02474200	-2.77057900
B	-2.85385100	1.33295900	-0.06152500
B	-0.27896100	2.68279700	1.44193600
B	-0.82287200	-1.66758300	-2.21920600
B	-1.78769700	-0.42218000	-2.61725900
B	-0.65408900	-1.34159100	2.34542900
B	1.59663200	-2.18738400	-0.86742400
B	-1.09237400	-2.39183500	1.03207900
B	2.08722400	1.01519900	-1.61972300
B	1.74016700	0.35189100	2.48022400
B	-2.35069000	0.67394800	1.36447600
B	0.60734100	1.27105600	-2.48537200
B	1.11048200	-1.13061500	2.26569100
B	-2.13376000	-1.08608300	1.48083200
B	-2.44199700	-2.02905600	0.16327600
Li	-0.49837300	3.91114600	-0.33832600
Li	0.46590000	-3.97743700	0.19864200
Li	3.94884900	0.44031200	-0.25029200
Li	-3.99707200	-0.51083900	0.11493500
H	5.33912900	1.21911700	1.29915900
H	5.82331200	0.71800900	1.02586500

H	5.34728500	0.06064500	-1.94690100
H	5.71116800	0.65175700	-1.66664800
H	-5.25667500	-0.44365300	1.96232900
H	-5.80499900	-0.66636300	1.50380800
H	-5.38486900	-0.96301200	-1.57933300
H	-5.91780400	-0.75763500	-1.09534800
H	-2.13028100	5.37667500	0.06528800
H	-2.03658100	5.51180000	-0.66493800
H	1.20710700	5.36096400	-0.51040600
H	0.67856000	5.89119700	-0.50729900
H	-0.77331100	-5.75860400	0.75092700
H	-0.67694500	-5.89883400	0.02206100
H	2.45785700	-5.01038900	0.14376200
H	2.07582100	-5.64515600	0.25117200

$(Li\text{-}3H_2)_4\&B_{36}$:

B	-2.21970500	-1.09874400	-1.30782500
B	0.24231300	-2.59619300	1.76850700
B	1.23888700	2.37712500	0.86661200
B	-0.94174200	1.33008100	2.31673900
B	2.38632700	-0.69891800	1.40745500
B	1.21229900	2.34524300	-0.91092900
B	2.34739700	-0.74784900	-1.37731800
B	-1.55533800	2.13403300	-0.86811900
B	0.19352900	-2.65475200	-1.60971100
B	2.25252800	1.07186500	1.37900000
B	-1.20578000	-2.40519700	-0.79416700
B	-0.20784500	2.56926000	-1.69559100
B	2.52485500	1.88179300	-0.03228700
B	0.83114700	1.46805500	2.28929300
B	-2.35187800	0.67221300	-1.33439300

B	-1.52724700	2.16126600	0.90936200
B	-1.00702000	1.25040900	-2.26123600
B	-1.62320900	-0.09601600	2.69675000
B	2.78682700	-1.50297900	0.02298700
B	1.58791300	-2.15952400	0.94102500
B	0.97553100	-1.35647600	-2.24325100
B	1.65565900	0.06971600	-2.62204500
B	-2.75199700	1.47410100	0.05022600
B	-0.15946000	2.62876000	1.68240500
B	-0.79718900	-1.49449700	-2.21686300
B	-1.69570500	-0.18811500	-2.57068300
B	-0.73269400	-1.41477100	2.36320100
B	1.56237400	-2.19038800	-0.83732600
B	-1.17761500	-2.36860400	0.98267900
B	2.21416800	1.02404100	-1.40766900
B	1.72962900	0.16149000	2.64246300
B	-2.31518700	0.72219700	1.45227900
B	0.76597600	1.38780100	-2.28889400
B	1.04063000	-1.27658800	2.33292200
B	-2.17902700	-1.05033700	1.48002000
B	-2.49037300	-1.90594100	0.10498200
Li	-0.29667300	3.98304300	-0.03346200
Li	0.32077600	-4.01037700	0.10010900
Li	4.03015900	0.30349400	-0.02435400
Li	-3.99950500	-0.32991500	0.10136000
H	5.37295000	2.51126200	-0.08012700
H	6.06371900	2.22503700	-0.04612100
H	5.46293100	-0.50397600	-1.56356800
H	5.82290200	0.14538700	-1.46843000
H	5.89718100	0.12240200	1.36336600

H	5.42019400	-0.40172300	1.60441900
H	-5.91736000	0.02624400	-1.16187700
H	-5.53524000	0.66659600	-1.22808300
H	-5.21659200	0.08971600	1.97175500
H	-5.78775100	-0.23798200	1.61577300
H	-5.33418500	-2.50443600	-0.09631500
H	-6.01779500	-2.20206600	-0.05791600
H	-1.47415700	5.35693800	1.36748100
H	-1.49030900	5.86942500	0.82176700
H	-0.91498400	5.37621900	-1.98618500
H	-1.00307700	6.02062200	-1.61517200
H	1.55540000	5.31133100	0.16846600
H	1.09397200	5.86247500	-0.04027800
H	-0.60811400	-5.51479000	1.54928300
H	-0.59483300	-6.02474500	1.00111400
H	2.37045100	-5.00985600	0.28880000
H	2.00917900	-5.65733500	0.18589300
H	-0.09834000	-5.54373200	-1.80246900
H	-0.03476700	-6.18304900	-1.41801100

$(Li\text{-}4H_2)_4\&B_{36}$:

B	-2.24650400	-1.13233000	-1.35752000
B	0.26554300	-2.55318800	1.71433600
B	1.12898600	2.43851300	0.78313600
B	-1.01601000	1.34692200	2.24933600
B	2.35886000	-0.60315200	1.33445600
B	1.09712800	2.39696600	-0.99455600
B	2.31125800	-0.66765100	-1.45322700
B	-1.66072600	2.11471600	-0.93716300
B	0.20316000	-2.62910800	-1.66319100
B	2.17732400	1.16321900	1.29816400

B	-1.19565400	-2.40544900	-0.84150000
B	-0.33218300	2.58289800	-1.77306600
B	2.42360800	1.96974600	-0.11909200
B	0.75197900	1.52872000	2.21365500
B	-2.42845500	0.63460100	-1.39442400
B	-1.63074700	2.15865700	0.84166100
B	-1.10082600	1.24129300	-2.32848100
B	-1.65607900	-0.09518300	2.63892000
B	2.77173000	-1.40358300	-0.04858600
B	1.59552300	-2.08575900	0.87867800
B	0.94944100	-1.31502800	-2.30769200
B	1.58993500	0.12615700	-2.69732800
B	-2.83857600	1.43483500	-0.00974700
B	-0.27037200	2.66156400	1.60476100
B	-0.81991500	-1.49690000	-2.27234500
B	-1.75429900	-0.21586000	-2.62771300
B	-0.73472700	-1.39304000	2.30803700
B	1.56212400	-2.12531200	-0.89980700
B	-1.16433900	-2.36768700	0.93594100
B	2.12710900	1.09898400	-1.48824400
B	1.68659100	0.24688700	2.56935700
B	-2.37505200	0.69843800	1.39286600
B	0.66887900	1.42351400	-2.36712500
B	1.03328000	-1.21089700	2.26973000
B	-2.19238900	-1.06789100	1.42929500
B	-2.49065000	-1.93844600	0.06086100
Li	-0.44106000	4.01599400	-0.11450400
Li	0.37404500	-3.98474300	0.05386500
Li	3.96032700	0.42716800	-0.10762800
Li	-4.02734100	-0.39602600	0.06327600

H	5.03962400	3.16850000	-0.22383700
H	5.78050200	3.25000700	-0.28831800
H	5.55680200	0.21294300	-1.65612800
H	5.61506700	0.95071000	-1.54245900
H	6.34513600	-1.84763300	-0.15092300
H	5.61222000	-1.97552200	-0.07441700
H	5.82311200	0.64511000	1.19270100
H	5.26965400	0.65770100	1.69677800
H	-5.63495700	-0.98635100	-1.38386200
H	-5.77559400	-0.25310000	-1.32716300
H	-5.26808900	-0.61046200	1.92725500
H	-5.83899100	-0.60663200	1.44298100
H	-5.08700200	-3.16397700	0.04463400
H	-5.82844300	-3.24933100	0.09511300
H	-5.68379300	1.97483400	0.06131500
H	-6.41541400	1.83959300	0.13900300
H	-0.07833300	5.61576800	1.63815000
H	-0.08659400	6.17191400	1.13696600
H	0.06104000	6.09240600	-1.30677200
H	0.04597300	5.54864100	-1.82132700
H	-2.30108200	5.54968900	-0.21076400
H	-2.62513800	4.88043000	-0.12586500
H	2.61622600	5.14256400	0.01589700
H	2.53617800	5.88076500	0.09813300
H	-0.15269600	-5.51629600	1.74525400
H	-0.16511900	-6.05850800	1.22902000
H	2.54823800	-4.85479400	0.17905400
H	2.22565700	-5.52446300	0.09204600
H	-2.60967700	-5.84553400	-0.23374400
H	-2.68791500	-5.10806000	-0.14349900

H	0.03449700	-5.58740000	-1.71040400
H	0.03927200	-6.14557200	-1.21136600

$(Li\text{-}5H_2)_4\&B_{36}$:

B	-2.23329000	-1.16805700	-1.36974600
B	0.32904200	-2.53695600	1.68526500
B	1.07536200	2.47546600	0.75961000
B	-1.03581100	1.33190700	2.23515000
B	2.37566100	-0.53963300	1.29728300
B	1.03426300	2.43631500	-1.01777100
B	2.31284400	-0.59929700	-1.48853300
B	-1.71642700	2.09152600	-0.94555500
B	0.24928200	-2.60767400	-1.69100600
B	2.15506100	1.22286100	1.26615400
B	-1.14975900	-2.41779600	-0.86152600
B	-0.40332800	2.59097400	-1.78770200
B	2.37551400	2.03751800	-0.15066600
B	0.72703600	1.55407400	2.18993200
B	-2.45198700	0.59501500	-1.40021500
B	-1.67760100	2.13333700	0.83366000
B	-1.14476500	1.23305000	-2.34127300
B	-1.64189300	-0.12468600	2.62552400
B	2.79978300	-1.32824400	-0.08928900
B	1.64276100	-2.03744300	0.84323800
B	0.96160600	-1.27520200	-2.33688900
B	1.56816100	0.18019300	-2.72763100
B	-2.87399900	1.38375400	-0.01270600
B	-0.32414100	2.66470400	1.58949500
B	-0.80247100	-1.49788800	-2.29193800
B	-1.76760000	-0.23765500	-2.63969500
B	-0.69375500	-1.40095700	2.28668600

B	1.60196000	-2.07572300	-0.93532300
B	-1.10960100	-2.38310100	0.91563400
B	2.09077500	1.16273100	-1.51975800
B	1.69225200	0.29295500	2.53782000
B	-2.38409200	0.65498000	1.38473700
B	0.62025000	1.45599800	-2.38958900
B	1.06963500	-1.17859300	2.23883900
B	-2.16296000	-1.10690000	1.41660600
B	-2.45051100	-1.98187200	0.04882100
Li	-0.53747700	4.01917100	-0.12496300
Li	0.46292700	-3.96472700	0.02055700
Li	3.95254400	0.53026800	-0.14964300
Li	-4.02862500	-0.47490200	0.06404000
H	5.09514000	3.13738600	-0.29511200
H	5.83600200	3.05478900	-0.36063600
H	5.43250900	0.16217300	-1.78353500
H	5.58554900	0.88617700	-1.66915200
H	6.44264700	-1.67071100	-0.03998000
H	5.71363300	-1.81822800	0.03777000
H	5.76451000	0.74629000	1.25308300
H	5.18297000	0.74229200	1.72532200
H	-5.60245800	-0.91262400	-1.48385800
H	-5.71544100	-0.17905600	-1.38606900
H	-5.13879000	-0.50373500	2.02651400
H	-5.75876000	-0.60170100	1.61747000
H	-5.19459400	-3.03606400	0.10135800
H	-5.93048500	-2.91933200	0.16990800
H	-5.75534300	1.91126500	0.13925700
H	-6.48690100	1.77510400	0.21733200
H	-0.20008600	5.61465300	1.63331000

H	-0.37934300	6.16936200	1.16304700
H	-0.09733300	6.09822900	-1.30613100
H	-0.07188500	5.55097900	-1.81706500
H	-2.42423300	5.52241700	-0.24370600
H	-2.74462600	4.85069200	-0.16176800
H	2.44561000	5.22180400	0.07808800
H	2.34159200	5.95564800	0.17197600
H	0.03395600	-5.50666000	1.71166100
H	0.06135400	-6.05043400	1.19712000
H	2.69967200	-4.76515400	0.07772200
H	2.38863400	-5.44026300	0.16746700
H	-2.36779400	-5.90317400	-0.28554300
H	-2.48016800	-5.17088900	-0.18890000
H	0.17622800	-5.56013100	-1.74083700
H	0.33760000	-6.11905800	-1.26901700
H	-8.00418500	-1.15300600	0.43604700
H	-7.89813600	-0.42611800	0.30916600
H	7.83582500	1.39342800	-0.01480200
H	7.91938000	0.66371600	-0.14275100
H	1.83219700	-7.83345400	0.03129400
H	1.16848700	-8.15230300	0.14757400
H	-1.11344000	8.15159500	-0.16275500
H	-1.83321600	7.99726300	-0.27987000

$(Li\text{-}6H_2)_4\&B_{36}$:

B	-2.19758200	-1.13896400	-1.32421200
B	0.34068800	-2.50772200	1.75058600
B	1.12367300	2.49442700	0.79967600
B	-1.00141900	1.37361100	2.27222100
B	2.40173000	-0.52556800	1.36098100
B	1.09086400	2.44631700	-0.97764600

B	2.35128100	-0.60050300	-1.42504300
B	-1.66205400	2.11876800	-0.91603800
B	0.27632600	-2.59749000	-1.62606200
B	2.19267900	1.23810900	1.31821000
B	-1.12525300	-2.39296400	-0.80404400
B	-0.34223400	2.60604800	-1.75492900
B	2.42502700	2.04271100	-0.10212400
B	0.76306800	1.58446500	2.23411000
B	-2.40564100	0.62496700	-1.36626800
B	-1.63086200	2.17015000	0.86280500
B	-1.08963900	1.24940500	-2.30453200
B	-1.61848700	-0.07700700	2.66815100
B	2.82695500	-1.32417800	-0.01937600
B	1.66150000	-2.02110300	0.91172500
B	1.00021700	-1.27307600	-2.27621500
B	1.61739500	0.17660600	-2.67212900
B	-2.82812000	1.42378800	0.01531900
B	-0.27828400	2.69857400	1.62220900
B	-0.76548400	-1.48398500	-2.23817600
B	-1.72034300	-0.21909700	-2.59767600
B	-0.67754800	-1.36181600	2.34134100
B	1.62834900	-2.06880600	-0.86669200
B	-1.09297000	-2.34827600	0.97311500
B	2.14116300	1.16247900	-1.46765300
B	1.71807100	0.31858200	2.59336600
B	-2.35022600	0.70022100	1.41946800
B	0.67675100	1.46085800	-2.34597800
B	1.08724200	-1.15050500	2.29963700
B	-2.14055100	-1.06247600	1.46219500
B	-2.42689500	-1.94287100	0.09796600

Li	-0.47558100	4.04682200	-0.10191800
Li	0.47291700	-3.94718600	0.09542500
Li	3.99586000	0.52686100	-0.08417000
Li	-3.99509700	-0.42683300	0.09475800
H	5.17695100	3.07134100	-0.20359200
H	5.91173000	2.94997400	-0.12697400
H	5.47031100	0.13104400	-1.71355800
H	5.62528800	0.85787900	-1.61924700
H	6.45578900	-1.71094200	-0.01251300
H	5.72851600	-1.85987200	0.07804400
H	5.79434300	0.69471500	1.35169100
H	5.21018500	0.65501200	1.81897000
H	-5.56099400	-0.93620200	-1.44276400
H	-5.69452300	-0.20442800	-1.35420300
H	-5.14444300	-0.42891500	2.03008800
H	-5.75029000	-0.51222100	1.59730700
H	-5.15191400	-3.01780100	0.28692900
H	-5.88988100	-2.91331800	0.21936400
H	-5.70476200	1.96254400	0.10778500
H	-6.43604800	1.81173500	0.06071600
H	-0.21403300	5.68144800	1.63338000
H	-0.24535400	6.23095600	1.12498700
H	-0.10903300	6.14893900	-1.30317200
H	-0.08694500	5.60167000	-1.81466700
H	-2.41456400	5.50634900	-0.18995200
H	-2.71979900	4.82630000	-0.11920700
H	2.48242100	5.20888500	0.03406500
H	2.36454600	5.94303000	0.11064800
H	0.07179000	-5.49834900	1.81639700
H	0.07246100	-6.04280900	1.30146300

H	2.68967700	-4.74543500	0.10842200
H	2.38231200	-5.42299100	0.19320300
H	-2.41688500	-5.83932500	-0.06975000
H	-2.52621500	-5.10355300	0.00359700
H	0.14838000	-5.57539200	-1.63362600
H	0.19189000	-6.12862100	-1.13017500
H	-7.95141100	-0.83281300	0.10615400
H	-7.98635500	-0.31446600	0.64133000
H	7.93263400	1.08745600	-0.24564600
H	7.96453300	0.55894700	0.27966900
H	2.06926300	-7.85093000	-0.05471800
H	1.41362700	-7.98011100	0.27595600
H	-1.43527800	8.16101200	-0.22013500
H	-2.11305700	7.86417800	-0.31164300
H	-1.12213800	-8.17302900	-0.17887100
H	-1.24875100	-8.05916100	0.54715800
H	7.32316300	2.85457700	-2.21405900
H	7.64549600	2.26167600	-2.53025000
H	1.09090800	8.28160000	0.29626900
H	1.19811300	8.17510700	-0.43403800
H	-7.68681000	-2.47153600	-2.08039400
H	-7.78046600	-2.84082600	-1.44011400
